# Phycocyanin Protects Against UVB-Induced Apoptosis Through the PKC α/βII-Nrf-2/HO-1 Dependent Pathway in Human Primary Skin Cells

**DOI:** 10.3390/molecules23020478

**Published:** 2018-02-22

**Authors:** Ki Mo Kim, Joo Young Lee, A-Rang Im, Sungwook Chae

**Affiliations:** 1 KM Convergence Research Division, Korea Institute of Oriental Medicine, 1672 Yuseong-daero, Yuseong-gu, Daejeon 34054, Korea; vsrc@kiom.re.kr (K.M.K.); jy0130@kiom.re.kr (J.Y.L.); lar747@kiom.re.kr (A.-R.I.); 2 Department of Korean Life Science and Technology, Korea University of Science and Technology, Daejeon 34113, Korea

**Keywords:** phycocyanin, heme oxygenase-1, nuclear factor erythroid-derived 2 (NF-E2)-like 2, apoptosis

## Abstract

Phycocyanin (Pc) is one of the active pigment constituents of *Spirulina* microalgae. It has been used for its potent antioxidant and anti-inflammatory properties. However, the protective effects of Pc against ultraviolet-B (UVB)-induced primary skin cells damage are still undefined. In the present study, we investigated whether Pc prevented UVB-induced apoptotic cell death in human dermal fibroblasts (HDF) and human epidermal keratinocytes (HEK). Pc induced the transcription of heme oxygenase-1 (HO-1). Furthermore, Pc treatments resulted in a marked increase in nuclear factor erythroid-derived 2 (NF-E2)-like 2 (Nrf-2) nuclear translocation. Also, Pc protected UVB induced apoptosis and reduced the p53 and Bax levels, as well as caspase-3 activation. Pc treatment showed a significantly enhanced effect on the phosphorylation of protein kinase C (PKC) α/β II, but not that of p38 mitogen-activated protein kinase (MAPK) or Akt. Induction of HO-1 induced by Pc was suppressed by Go6976, a selective inhibitor of PKC α/β II. In addition, knockdown of HO-1 by small interfering (siRNA) caused a significant increase in poly (ADP-ribose) polymerase 1 (PARP-1) cleavage and caspase-3 activation after Pc pretreatment. Taken together, our results demonstrate that Pc-induced expression of HO-1 is mediated by the PKC α/β II-Nrf-2/HO-1 pathway, and inhibits UVB-induced apoptotic cell death in primary skin cells.

## 1. Introduction

Phycocyanin (Pc), a major pigment constituent of the phycobilisomes in *Spirulina*, has been suggested to exhibit radical scavenging properties [1], to reduce inflammatory responses [2,3], and reduce oxidative stress [1,4]. This phycobili protein enhances wound healing [5], retards platelet aggregation [6,7] and acts as a photodynamic agent to eradicate cancer cells in vitro [8,9]. Although the pharmaceutical effects of Pc have been reported, the mechanism underlying its anti-apoptotic effects is still poorly defined.

Ultraviolet radiation (UV) is divided into UVC (200–280 nm), UVB (280–320 nm), and UVA (320–400 nm). UVB is physiologically significant, penetrating into the papillary area of the dermis and inducing DNA damage in residing dendritic cells (DC) [10,11,12], and keratinocytes. These cells are perturbed both phenotypically and functionally, undergoing apoptosis upon exposure to UVB. Apoptosis can be induced in the region that suffers the greatest exposure, and surrounding cells can be partially damaged [10,12]. Apoptosis is the process of programmed cell death, which involves a series of morphological changes, including cell detachment, cell shrinkage, chromatin condensation and DNA fragmentation. This process is controlled by the balance between pro-apoptotic and anti-apoptotic signaling pathways [13].

Heme oxygenase-1 (HO-1) is the rate-limiting enzyme in the oxidative degradation of heme into bilirubin, iron, and carbon monoxide (CO). HO-1 has anti-inflammatory ability and is an important molecule in the host defense against oxidative stress [14]. HO-1 deficient mice exhibit a phenotype of an increased inflammatory state [15]. Exogenous administration of HO-1 by gene transfer protected lung cells from free radical-induced lethality and exhibited anti-inflammatory effects in the lungs [16]. It is important to substantiate this interesting property of the phytochemical Pc or other phytochemicals. There are several reports regarding HO-1 induction by Pc [17,18]. In the transcriptional regulation of antioxidant proteins, HO-1 and NAD(P)H:quinone oxidoreductase (NQO-1) are regulated by nuclear factor erythroid-derived 2 (NF-E2)-like 2 (Nrf-2), which is repressed by its negative regulator kelch-like ECH-associated protein 1 (Keap1) [19].

It has been reported that activation of Nrf-2 and the subsequent transcription of Nrf-2-related antioxidant proteins confers photoprotection response by inhibiting pro-inflammatory cytokines [20]. Nrf-2 protein might be phosphorylated by several signal transduction pathways, including the mitogen-activated protein kinase (MAPK), phosphoinositide 3-kinase (PI3K)/Akt, protein kinase C (PKC), and extracellular signal-regulated kinase (ERK) pathways [21]. Several studies have suggested that the mechanisms of HO-1 induction involve pathways of MAPK, PI3K/Akt and Nrf-2 pathways [22,23,24]. Nrf-2 is known to induce expression of HO-1, and the inhibitory effect of HO-1 on UVB-mediated apoptotic cell death has been suggested earlier in UV-irradiated mice in which the inhibition of HO-1 enzyme activity by the substrate antagonist protoporphyrin-tin (SnPP) was found to increase epidermal apoptotic cell numbers and upregulation of nuclear Nrf2 [25,26].

We hypothesized that Pc induces HO-1 expression via the Nrf-2 pathway in primary skin cells to protecting against UVB-mediated apoptosis. Thus, in this study, we determined whether Pc regulates the Nrf-2/HO-1 activation and whether the cytoprotective effect of Pc on the UVB-induced cell death requires HO-1 expression in human dermal fibroblasts (HDF) and human epidermal keratinocytes (HEK). We further explored whether Pc exerts its cytoprotective effect against UVB-induced cell death via the PKC α/β II dependent activation of the Nrf-2/HO-1 signaling pathway.

## 2. Results

### 2.1. Pc-Induces HO-1 mRNA and Protein Expression without Cytotoxicity in Human Primary Skin Cells

**Figure 1:**
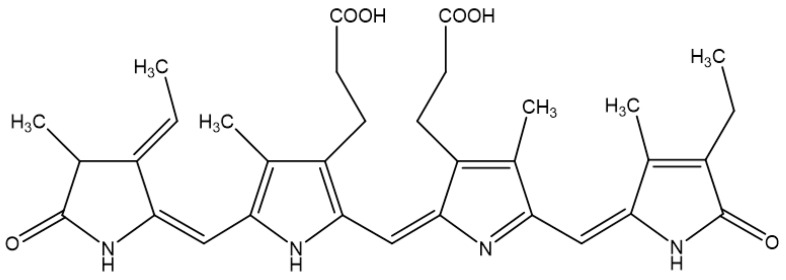
Chemical structure of phycocyanin (Pc).

**Figure 2:**
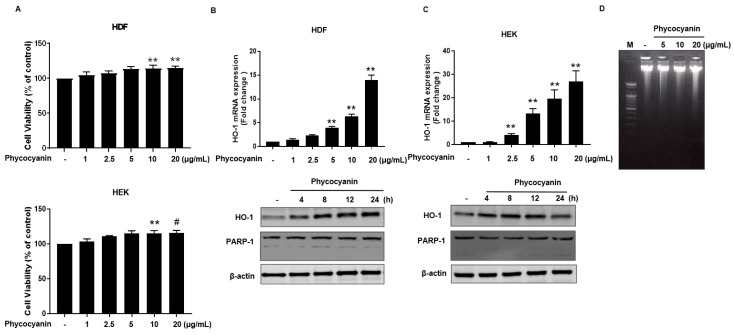
Effect of Pc on cell viability and HO-1 expression in primary skin cells. (**A**) HDF and HEK cells viabilities were assessed using the (3-(4,5-dimethylthiazol-2-yl)-2,5-diphenyltetrazolium bromide) MTT assay. Cells were incubated with Pc at the indicated concentration for 24 h intervals, followed by incubation with MTT solution for 2 h. Each value is expressed as mean ± standard deviation (SD) (*n* = 3); (**B**) Total RNAs was extracted from HDF after dose-dependent treatment with Pc for 8 h. real time-quantification polymerase chain reaction (RT-qPCR) was performed with the HO-1 primers listed in the ‘Materials and Methods’ (top panel). Indicated time-dependent treatment with Pc (low panel). Expression of HO-1, poly (ADP-ribose) polymerase-1 (PARP-1), and β-actin were detected by western blotting. Each value is expressed as mean ± SD (*n* = 3); (**C**) HEK cells were treated with different concentration of Pc for 8 h (mRNA level) or treated with Pc for different times (protein level). Expression of HO-1, PARP-1, and β-actin were detected by western blotting; (**D**) HEK cells were treated with different concentration of Pc for 24 h DNA fragmentation analysis was performed with the ‘Material and Methods’ Data were obtained from three independent experiments and are expressed as the means ± SD, ** *p* < 0.01 versus the respective control groups. HO-1, heme oxygenase-1; HDF, human dermal fibroblasts; HEK, human epidermal keratinocytes.

The molecular structure of Pc is shown in Figure 1. We first examined whether Pc is cytotoxic, and found that Pc is not cytotoxic since doses up to 20 μg/mL did not alter the cell viability in HDF and HEK (Figure 2A). We next assessed whether Pc can induce HO-1, poly (ADP-ribose) polymerase-1 (PARP-1) expression in primary skin cells. Pc increased HO-1 mRNA and protein levels in a concentration-dependent and time-dependent manner in both HDF and HEK. However, Pc did not show any effect on PARP-1 cleaved form (Figure 2B,C, respectively). In addition, we examined DNA protection by Pc. These was no significant difference between the control and Pc-treated cells (Figure 1D). These results indicate that Pc-induced HO-1 expression at the mRNA and protein levels in human primary skin cells without cytotoxicity and Pc alone did not induce a typical apoptotic response in HEKs.

### 2.2. Pc-Induced HO-1 Expression Is Mediated by Nrf-2

**Figure 3:**
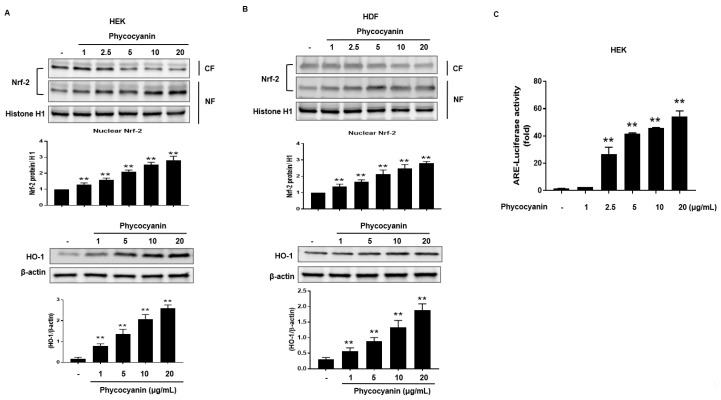
Pc-induced expression of HO-1 is mediated by Nrf-2. (**A**) HEK cells were treated with different concentrations of Pc for 6 h, and then nuclear fractions (NF) and cytosolic fractions (CF) were prepared and analyzed by western blotting analysis (upper panel). HEK were treated with different concentration of Pc for 24 h. Expression of HO-1 and β-actin was detected by western blotting (lower panel); (**B**) HDF cells treated with different concentration of Pc for 6 h, and then nuclear fractions (NF) and cytosolic fractions (CF) were analyzed by western blotting analysis (upper panel). HDF were treated with varying concentration of Pc for 24 h. (low panel). Expression of Nrf-2, HO-1, and β-actin were detected by specific antibodies (lower panel); (**C**) HEK cells were transfected with plasmid DNA (ARE-luciferase construct). Cells were allowed to recover for 24 h. Subsequently, cells were treated with different concentration of Pc for 6 h, and subjected to luciferase assays. Data were obtained from three independent experiments and are expressed as the means ± SD, ** *p* < 0.01 versus the respective control groups. Nrf-2, nuclear factor erythroid-derived 2 (NF-E2)-like 2; ARE, antioxidant response element.

Nrf2 translocates to the nucleus where it interacts with the antioxidant response element (ARE) to induce ARE-mediated antioxidant genes, including HO-1 [27]. Therefore, we attempted to examine the nuclear accumulation of Nrf-2 protein in Pc-stimulated primary skin cells. The nuclear levels of Nrf-2 were increased by treatment with Pc in a concentration-dependent manner while the cytosolic Nrf-2 was decreased. (Figure 3A,B upper panel). In addition, Pc treatment significantly increased expression of HO-1 (Figure 3A,B lower panel). Additionally, a luciferase reporter gene assay was performed in HEK. Cells were transfected with luciferase cDNAs under transcriptional control of an ARE. As a result, Pc was shown to significantly activate ARE-mediated transcriptional (Figure 3C). This indicates that the Pc activated both the Nrf2/ARE pathway system.

### 2.3. Pc Protects against UVB-Induced Apoptotic Cell Death in Primary Skin Cells

**Figure 4:**
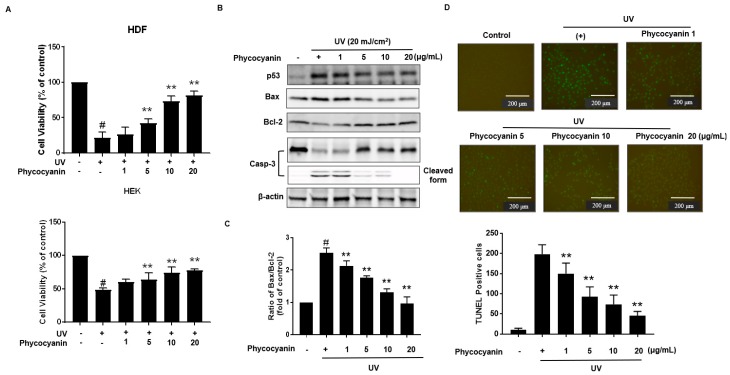
Cytoprotective effect Pc against UVB-induced apoptosis. (**A**) HDF and HEK cells were pretreated with different concentrations of Pc for 24 h and then washed with phosphate-buffered saline (PBS). In PBS, the cells were exposed to UVB (20 mJ/cm^2^) for 20 min, and then incubated in normal medium for 2 h. Viability of cells under different treatments was examined by an MTT assay and is expressed as a percentage of the control cells; (**B**). Cells were preincubated with the indicated concentration of Pc for 24 h and then washed with PBS. The cells were exposed to UVB radiation (20 mJ/cm^2^) in PBS for 20 min, and then incubated in normal medium for 2 h. Western blotting analysis using anti-p53, Bax, Bcl-2, caspase-3-specific antibodies was performed; (**C**) HEK cells were pretreated with the Pc (20 µg/mL) for 24 h, exposed to UVB light (20 mJ/cm^2^) for 20 min, and then incubated for 4 h. Apoptotic cells were detected by the TUNEL assay and quantified. Each value is expressed as mean ± SD (*n* = 3). ^#^ *p* < 0.05 versus the control groups. ** *p* < 0.01 indicates significant difference versus UVB light.

Primary keratinocytes were pretreated with varying concentration of Pc followed by treatment with or without UVB (20 mJ/cm^2^). Cells viability was assessed using the MTT (3-(4,5-dimethylthiazol-2-yl)-2,5-diphenyltetrazolium bromide) assay; Pc pretreatment was shown to markedly protect the UVB-exposed cells (Figure 4A). Additionally, we analyzed the expression of p53, Bax, Bcl-2, and caspase-3 in the keratinocytes cells. In our results, Pc significantly decreased p53, Bax/Bcl-2 expression levels, and Pc suppressed the activation of caspase-3 compared with that in cells exposed to UVB only (Figure 4B,C). Apoptotic chromatin condensation and DNA fragmentation (as indicated by terminal deoxynucleotidyl transferase dUTP nick end-labeling (TUNEL) staining, which were increased by UV exposure, were blocked by the addition of Pc (Figure 4D). These results indicate that Pc exhibited a protective effect against UVB-induced apoptotic cell death.

### 2.4. Induction of HO-1 and Activation of Nrf-2 by Pc via Phosphorylation of PKC α/β II

**Figure 5:**
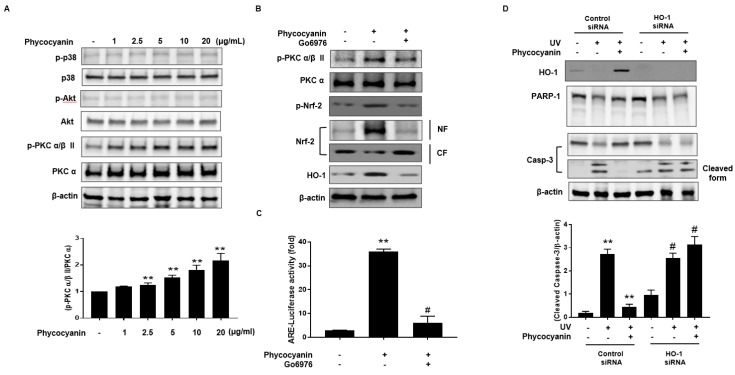
Pc activates HO-1 expression through a protein kinase C (PKC) α/β II-dependent pathway in primary skin cells. (**A**) HEK cells were treated with varying concentration of Pc for 4 h. Cell lysates were analyzed by western blotting. Band densities of phospho-PKC α/β II were normalized to those of total PKC α; (**B**) Cells were preincubated for 30 min with Go6976 (2 μM) and then treated with Pc (20 µg/mL) for 12 h, and then NF and CF were analyzed by western blotting; (**C**) Cells were transfected with control siRNA or HO-1 siRNA, and then cells were allowed to recover for 24 h. The transfected cells were preincubated with Go6976 (2 μM) for 1 h, and then treated with Pc for 18 h, subjected to luciferase assays; (**D**) Cells were transfected with control siRNA or HO-1 siRNA for 12 h. The transfected cells were preincubated with Pc (20 µg/mL) for 24 h, exposed to UVB light (20 mJ/cm^2^) for 20 min, and then incubated for 2 h. Cell lysates were analyzed by western blotting. Data shown in the graphs are expressed as means ± SD of three independent experiments. ** *p* < 0.01 versus the control groups. ^#^ *p* < 0.05 versus the Pc-treated groups.

To further elucidate the upstream signaling pathway involved in Pc-mediated Nrf-2 activation and induction of HO-1, we examined the effect of Pc on p38, Akt and PKC α/β II. Phosphorylation of p38 and Akt was not detected in cells treated with Pc. However, Pc treatment was able to enhance the phosphorylation of PKC α/β II (Figure 5A). We also, confirm the involvement of PKC α/β II in the Nrf2 nuclear translocation for Pc induced HO-1 expression. We tested the phosphorylation pattern of PKC α/β II and Nrf2, nuclear translocation of Nrf2, and HO-1 expression after pretreatment with Go6976, a selective inhibitor of PKC α/β II. Inhibition of PKC α/β II activity significantly suppressed the phosphorylation and nuclear translocation of Nrf2, and HO-1 expression, induced by Pc treatment (Figure 5B). We further confirmed the activation of ARE after treatment with Pc. Pc treated with increased ARE transcriptional element, however pretreatment with Go6976 was decreased ARE-luciferase activity (Figure 5C). In addition, we further examined the change in HO-1 expression with transfection of HO-1 small interfering RNA (siRNA). Pc protected against UVB-induced PARP-1 (apoptosis marker) and caspase-3 cleavage, in cells transfected with control RNA, but not in cells transfected with HO-1 siRNA (Figure 5D). These results suggest that Pc modulates HO-1 induction and Nrf-2 nuclear translocation via the PKC α/β II pathway, resulting in anti-apoptotic activity.

## 3. Discussion

Previous research has shown that Pc (Figure 1) exhibits antioxidative, anti-inflammatory, hepatoprotective and neuroprotective effects that have been demonstrated both in in vitro and in vivo studies [28,29,30,31,32], at least in part by providing energy repletion to UV-irradiated cells to ameliorate apoptosis and cellular energy loss [33,34]. However, the precise molecular mechanisms underlying the cytoprotective and anti-apoptotic effects remain largely unknown. The present study was undertaken to investigate the anti-apoptotic effects of Pc, and its underlying mechanisms. We found that induction of HO-1 expression by Pc resulted from transcriptional activation (Figure 2). Furthermore, Pc was able to significantly increase the nuclear accumulation of Nrf-2 and ARE-mediated transcriptional activity (Figure 3). 

UVB irradiation triggers cytotoxic damage to the skin, which interferes with the normal cellular function, and finally culminates in photodamage, photoaging, and photocarcinogenesis [35]. Furthermore, UVB is known to be the most potent mutagenic component causing direct damage to cellular DNA as well as production of reactive oxygen species (ROS) in the epidermis, dermis [36,37]. Indeed, UVB represent carcinogenesis occurs via mutagenic DNA modification in susceptible cells due to insufficient repair and pathological enforcement of survival pathway and/or cell death pathway. Particularly, the role of UVB in skin photo damages, including skin cancer, has been widely reported because of the increasing incidence of photo damages [38]. Furthermore, the apoptotic response directly indicates the degree of genetic damage. We assessed whether Pc could inhibit UVB-induced skin cells apoptosis. Cell viability and western blotting analysis further showed that Pc increased the expression of anti-apoptotic factors, such as Bcl-2, in the UVB-exposed cells, while simultaneously decreasing the expression of the pro-apoptotic factors, p53, Bax, and caspase-3 (Figure 4A,B). Moreover, TUNEL staining showed that Pc significantly decreased DNA fragmentation in the UVB-irradiated cells (Figure 4C).

The PI3K/Akt and MAPK pathways also have been reported to be involved in HO-1 expression [39,40,41] and in Nrf-2-dependent transcription [41]. PKC also phosphorylates Nrf-2 in the Keap1-interacting domain [42,43]. A correlation between ERK activation and Nrf-2-mediated antioxidant enzyme expression has been reported [44]. Our results suggest that the increased expression of HO-1 and nuclear translocation of Nrf-2 by Pc are associated with of PKC α/β II, but not p38, or AKT, phosphorylation in keratinocytes (Figure 5A). In addition, the selective inhibitor of PKC α/β II, Go6976, suppressed Pc-induced HO-1 expression (Figure 5B). Using siRNA knockdown of HO-1 expression, Pc inhibition of UVB-induced PARP-1 cleavage, was partially, but significantly, reversed (Figure 5C). Nrf-2, a bZIP transcription factor, is an essential ARE-binding factor involved in both constitutive and inducible expression of glutathione biosynthetic enzymes [45]. It has been proposed that Keap1 and Nrf-2 constitute a crucial sensor for oxidative stress and mediate a key step in the signaling pathway, leading to transcription activation by the Nrf-2 nuclear shuttling mechanism [46], and cytoprotective role of the Keap-1-Nrf2 pathway [47]. Our data show that Pc activates Nrf-2 translocation and ARE-mediated transcription activation in skin cells (Figure 3). 

Nrf-2 has emerged as a promising molecular target for the pharmacological prevention of skin damage caused by UV exposure. Although UVB induced apoptotic cells death was suppressed by Pc, cell cycle arrest, lipids signaling pathway remain unclear. Our findings show that Pc significantly improves cells viability in UVB-irradiated primary skin cells, and suppresses that apoptosis activated by UVB (Figure 4). In addition, Pc-induced HO-1 expression suppresses UVB-induced PARP-1 cleavage, and caspase-3 activation (Figure 5). These results suggested that Pc is able to attenuate UVB-induced apoptotic cell death in human dermal keratinocytes. Notably, the present study confirmed that these were mediated at least in part, via inducing PKC α/β II/Nrf-2-mediated HO-1 pathway. Further studies are required to investigate the in vivo anti UV effects of Pc.

## 4. Material and Methods

### 4.1. Reagents 

Pc was purchased from Sigma-Aldrich, Inc. (St. Louis, MO, USA). Go6976, was purchased from Calbiochem Company (San Diego, CA, USA). HO-1 and control siRNAs were obtained from Santa Cruz Biotechnology (Dallas, TX, USA) and antibodies to p-PKC α/β II (#9375), p-Akt (#2971), PARP-1 (#9542), p-p38 (#9212), cleaved caspase-3 (#9661), and β-actin (#4967) were obtained from Cell Signaling Technology (Beverly, MA, USA). p-Nrf-2 (ab76026) was purchased from Abcam (Cambridge, UK). Nrf-2 (sc-722), histone-H1 (sc-393358), procaspase-3 (H-277), and HO-1 (sc-10789) antibodies were purchased from Santa Cruz Biotechnology. All other reagents were purchased from Sigma-Aldrich unless otherwise indicated. Pc solutions was prepared in dimethyl sulfoxide (DMSO) and stored at −20 °C.

### 4.2. Primary Cell Culture

Primary HDF were obtained from ScienCell Research Laboratories (Carlsbad, CA, USA). HDF cells were propagated in fibroblast medium supplemented with 5% fetal bovine serum (FBS), 1% fibroblast growth supplement, and 1% penicillin and streptomycin (P/S). HEKs were purchased from Lonza (Walkersville, MD, USA) and maintained in KGM-Gold SingleQuots^TM^ was purchased from Lonza medium kit containing supplements and growth factors. Both primary cultures were maintained in a humidified 5% CO_2_ incubator at 37 °C. HDF and HEKs cells were purchased from each company are used for research application by informed consent or legal authorization. Their cells used passages 6. Viabilities of HDF and HEK were assessed by an MTT assay as previously described [48].

### 4.3. UVB Radiation

Cells were pre-incubated with the indicated concentration of Pc for 24 h, washed with PBS and exposed to a UVB (20 mJ/cm^2^) light source using a UVP Cross linker (Ultra-Violet Products Ltd., Cambridge, UK) for 20 min Afterwards, the cells were washed with PBS, medium was added, and cells were further incubated for 2 h.

### 4.4. RNA Interference

Small interfering RNAs (siRNA) against HO-1 and control siRNA (Santa Cruz Biotechnology) were transiently transfected into cells using Lipofectamine 2000 according to the manufacturer’s instructions (Invitrogen, Carlsbad, CA, USA). Aliquots of 1 × 10^5^ cells/well were plated in 6-well plates on the day before transfection and grown to approximately 70% confluence. The cells were then transfected with 100 nmol of HO-1 siRNA or control siRNA plus 100 pmol of Lipofectamine for 6 h in Opti-MEM^®^I reduced serum medium (Invitrogen). Following an incubation period of 24 h, the protein levels were measured by western blot analysis.

### 4.5. DNA Fragmentation Assay

Pc was indicated concentration added, and the cells were incubated for 24 h. The cells were re-suspended in lysis buffer (20 mM Tris-HCl, pH 8.0, 10 mM, ethylenediaminetetraacetic acid (EDTA), 0.2% Triton X-100) containing 0.1 mg/mL proteinase K and 50 μg/mL RNase A, and the samples were incubated at 37 °C overnight. The DNA was subjected to electrophoresis using a 1.5% agarose gel. The agarose gel was stained with 1 μg/mL ethidium bromide in 0.5X tris-borate-EDTA (TBE) buffer to visualize the DNA fragmentation, and the gel was examined using photographs taken under UV light.

### 4.6. Quantitative Reverse Transcription PCR (RT-qPCR)

Total cellular RNA was extracted using Trizol reagent according to the manufacturer’s instructions (Thermo Fisher Scientific, Waltham, MA, USA). To quantify the HO-1 gene expression in HDF and HEK cells were using the TaqMan Primers (Hs01110250_ml), RT-qPCR was performed with TaqMan^®^Universal PCR Master Mix (Applied Biosystems, Waltham, MA, USA) using the ABI QuantStudio^TM^ 6 Flex Real-Time PCR system (Applied Biosystems). The relative mRNA expression levels of HO-1 was normalized to that of β-actin. Each sample was assayed in triplicate and relative mRNA expression levels were calculated using the relative quantitation comparative CT method (ΔΔCt method).

### 4.7. Preparation of Cytosolic and Nuclear Fractions 

Cytosolic and nuclear fractions were prepared using NE-PER^TM^ Nuclear and Cytoplasmic Extraction Reagent Kit (Thermo Fisher Scientific). Briefly, cells were trypsinized and suspended in cell fractionation buffer for 5 min. The suspension was centrifuged at 500× *g* for 5 min at 4 °C and the supernatant containing the cytoplasmic fraction was collected. The pellet containing the nuclear fraction was re-suspended in ice-cold cell disruption buffer, vortexed, and incubated for 5 min on ice to ensure complete nuclear protein extraction. Nuclear Nrf-2 was detected by western blotting.

### 4.8. Western Blotting 

After, different treatments, the cell lysates were prepared from HDF and HEK (5 × 10^6^) in 1× Laemmli lysis buffer (2.4 M glycerol, 0.14 M Tris-HCl (pH 6.8), 0.21 M sodium dodecyl sulfate (SDS), and 0.3 mM bromophenol blue), and boiled for 10 min. The protein content was measured using the BCA Protein Assay Reagent (Pierce, Waltham, MA, USA). Protein samples (20 μg) were diluted with 1× lysis buffer, separated by SDS-polyacrylamide gel electrophoresis, and transferred onto polyvinylidene fluoride membranes (PVDF). The membranes were then incubated with primary antibodies against Nrf-2 (1:1000), HO-1(1:1000), histone H1 (1:1000), p53 (1:1000), Bax (1:1000), Bcl-2 (1:1000), procaspase-3 (1:1000), cleavage caspase-3 (1:1000), and β-actin (1:1000). Subsequently, the membranes were washed, incubated with horseradish peroxidase-conjugated secondary antibodies, washed again, and detected using an enhanced chemiluminescence detection system from Amersham Bioscience (Buckinghamshire, UK). Protein expression levels were determined by analysis of the signals that were captured using an image analyzer (Las-3000, Fujifilm, Tokyo, Japan).

### 4.9. ARE-Luciferase Assay

The effects of Pc on ARE activity were assayed using a Luciferase Reporter Assay System (SABiosciences, Frederick, MD, USA). Briefly, HEK cells were seeded at a density of 1 × 10^5^ cells/well in 6-well plates and grown to 60–70% confluence. Cells were then transfected with 100 ng of ARE luciferase reporter construct using Lipofectamine^TM^ 2000 transfection reagent according to the manufacturer’s protocol (Invitrogen). Renilla-CMV-Renilla luciferase was used to correct for variations in transfection efficiency. Twelve hours after transfection, cells were treated with the Pc for 24 h. After incubation, cells were lysed was carried out using the reporter lysis buffer. Cell extracts were then mixed with a luciferase substrate (Promega, Madison, WI, USA), and luciferase activity was measured using a Tristar LB 941 multimode microplate reader (Tristar, Berthold, Wildbad, Germany).

### 4.10. Evaluation of Apoptotic Cell Death

HEK cells were plated at 1 × 10^5^ cells/mL in chamber slides. Cells were pretreated with Pc for 24 h, washed with PBS and then exposed to a UVB radiation (20 mJ/cm^2^) for 20 min Afterwards, the cells were washed with PBS, medium was added, and cells were further incubated for 4 hours. And found to induce apoptosis was assessed by a fluorescence TUNEL assay kit according to the manufacturer’s recommendations (Roche-Applied Science, Indianapolis, IN, USA). Images were captured using an inverted-fluorescence microscope (Olympus IX71, Tokyo, Japan). At least 100 TUNEL-positive cells were scored for each experiment performed in triplicate. 

### 4.11. Statistical Analysis 

The data are presented as the means ± standard deviations (SD) of at least three separate experiments. Comparisons between two groups were made using the Student’s *t*-test provided in the Graph Pad software version 5 (Graph Pad Software, La Jolla, CA, USA); significance was established at *p* < 0.05.
